# Understanding the Interactions between Small-Scale Fisheries and the Mediterranean Monk Seal Using Fishermen’s Ecological Knowledge

**DOI:** 10.3390/ani13132164

**Published:** 2023-06-30

**Authors:** Marios Papageorgiou, Anastasis Karonias, Athena Eftychiou, Louis Hadjioannou

**Affiliations:** Enalia Physis Environmental Research Centre, Acropoleos 2, Aglantzia, Nicosia 2101, Cyprus; pro.atk.1993@gmail.com (A.K.); athena.eftychiou99@gmail.com (A.E.); l.hadjioannou@enaliaphysis.org.cy (L.H.)

**Keywords:** *Monachus monachus*, depredation, incidental capture, marine mammals-fisheries interactions, fisheries management, Levantine Sea, Eastern Mediterranean

## Abstract

**Simple Summary:**

Marine mammals are known to interact with fisheries worldwide, usually in the form of depredation which is the act of removing captured fish or bait from fishing gear. Depredation can significantly damage fishing gear and catch, thus increasing operating costs. Additionally, such interactions may lead to the incidental capture of marine mammals with consequences that may lead to injury and death. The endangered Mediterranean monk seal has been known to interact with small-scale fisheries in the Mediterranean Sea, with consequent negative impacts on the species and fishermen. While the population of the species has steadily grown to about 20 individuals in Cyprus in the past 12 years, the Mediterranean monk seal is currently facing different anthropogenic stressors. Interviews were conducted with 90 fishermen with the aim to get a better understanding of the interactions between the Mediterranean monk seal and small-scale fisheries in the Republic of Cyprus. The findings indicate minimal interactions between the monk seals and fisheries, but it is likely that the species may accidentally become entangled on fishing gear, potentially leading to adverse consequences and mortality. This study proposes specific area- and time-based protected areas as a management action to mitigate against these interactions.

**Abstract:**

Interactions between fisheries and marine mammals have been well documented in almost all existing fishing gears around the world, often associated with detrimental consequences. Interactions of the endangered Mediterranean monk seal (*Monachus monachus*) with small-scale fisheries have been previously documented in the Mediterranean; this is a problem that seems to be growing in recent years. The present study aims to understand for the first time the nature and extent of interactions between the Mediterranean monk seal and small-scale fisheries in the Republic of Cyprus. The data were collected by conducting in-person semi-structured interviews, between November and December 2020, with 90 fishermen operating from nine different ports, extending throughout the entire coastline of the Republic of Cyprus. The results revealed minimal interactions between the Mediterranean monk seals and small-scale fisheries. The findings indicate that interactions are more likely to occur at depths of less than 10 m, closer to the coast, with the use of trammel nets and gillnets, and during the spring and summer months. The encounter, depredation and incidental capture rates were calculated at 0.01 (0.95%), 0.005 (0.51%) and 0.0004 (0.04%) per fisher, respectively. Spatiotemporal closed areas are proposed as a potential solution to mitigate these interactions.

## 1. Introduction

The Mediterranean monk seal *Monachus monachus* has been recently re-categorised from ‘Critically Endangered’ to ‘Endangered’ on the IUCN Red List global assessment, due to the increase in population size over the last few years. The global species population is estimated as being less than 700 individuals, of which approximately 350–450 are adult individuals [1]. Today, the largest subpopulations of the Mediterranean monk seal in the Mediterranean Sea are found along the coasts of mainland Greece and at the islands of the Ionian and Aegean seas, in southern and western Turkey and Cyprus [2,3,4,5,6,7]. There have also been some sporadic sightings of individual monk seals in Syria [8], Lebanon [9], Israel [10], Egypt [11], Libya [12], Spain [13], Italy [14], Croatia [15] and Albania [16]. More recently, sightings of monk seals have also been recorded along the Israeli coast [17]. 

The species was first officially reported in Cyprus in 1959 [18] and since then the population steadily declined [19]. Field surveys conducted in 1997 [20] and over the period 2005 to 2006 identified suitable habitat for the species but did not record any reproductive activity [21]. Up until 2009, the species was considered as being close to extinction [5,7]. According to stories from locals, in the 1940s and until the 1960s, new-born pups usually younger that one-month-old were captured by fishermen and displayed to the public upon a fee as a form of entertainment, calling the crowd to see the ‘little mermaid’. While this practice generated additional income for the fishermen and locals at the time, it most likely had a profound impact on the species’ population size and dynamics [22]. However, a study conducted between 2009 and 2018 indicated a very promising future for the species [7]. Specifically, since 2009, the number of sightings of the Mediterranean monk seal increased with most recorded sightings being juveniles and adults (95%) and only 5% of sightings being of newborn pups. The study also identified 17 suitable monk seal shelters along a 370 km stretch of coastline [7]. The increased number of sightings of pups, juveniles and adults over the last years and the suitability of the habitats on the island indicates that there is a presently permanent population of the Mediterranean monk seal in Cyprus [7].

Interactions between fisheries and marine mammals have been reported worldwide, often causing some serious and adverse effects on the conservation of certain species, e.g., [23,24,25]. These interactions are primarily related to the act of depredation, i.e., the removal of captured fish or bait by a predator, by marine mammals often causing them to unintentionally become caught on fishing gear [26]. Depredation can significantly reduce the value of the catch, damage fishing gear and significantly increase operating costs, while also increasing the chances of species’ accidental entanglement [27]. An example is the Yangtze River dolphin (*Lipotes vexillifer*) which was brought to extinction as a result of incidental capture in fisheries [28]. Currently, the vaquita (*Phocoena sinus*) [29] and the Yangtze finless porpoise (*Neophocaena asiaeorientalis asiaeorientalis*) [30] are in the same fate if no immediate conservation measures are taken. Accidental entanglement of the Mediterranean monk seal on fishing gear due to depredation has been reported in Greek fisheries and has been identified to be among the main causes of the species’ mortality [31,32,33]. Accidental entanglement may lead to death by drowning or from deliberate killing by angry fishermen as an act of revenge due to the damages caused on fishing gear and catch [31,33].

Overfishing as a result to meet global demand for fish, unselective and destructive fishing methods and poor management has led to the depletion and collapse of several fish stocks worldwide [34,35,36]. It is likely that interactions between fisheries and marine mammals will continue to grow as the competition for the same biological resources is increasing and prey availability for marine megafauna is decreasing [37]. Interactions between fisheries and marine mammals in Cyprus have been previously reported for small-scale [38] and pelagic longline fisheries [39], with reports of incidental capture and mortality. Improving our understanding of these interactions could lead to better management and conservation actions which have the potential to eventually reduce the conflicts between fisheries and marine megafauna, with mutual benefits. Following an interview-based approach, this study aims to investigate for the first time the nature and extent of interactions between the Mediterranean monk seal and small-scale fisheries (SSF) in the Republic of Cyprus and to explore potential conservation actions that could mitigate SSF-monk seal conflicts. 

## 2. Materials and Methods

In-person interviews were conducted with 90 active professional small-scale fishermen from nine main fishing ports from the regions of Famagusta, Larnaca, Limassol and Paphos between November and December 2020. The interviews covered 30% of the total professional small-scale fishermen across all coastal regions of the Republic of Cyprus. Participants belonged to the fleet segment consisting of the licensed categories A and B. Licensed boats of categories A and B are considered the professional small-scale fishing vessels, most of which have a length size of 6–<12 metres and are allowed to operate every day all year-round following restriction measures on the fishing gears used and landing sizes, according to the national and community law (Basic Fisheries Law Cap. 135 and subsequent amendments of 1961 to 2022; Fisheries Regulations of 1990 to 2023 based on Article 6 of the Basic Fisheries Law). The differences between the two categories lie only in the fishing gear allowed for use, where licensed boats of Category A are allowed to use a maximum of 4000 m of nets and Category B a maximum of 3000 m of nets. The small-scale inshore fleet is composed of wooden boats that operate mostly using trammel nets, set gillnets and set bottom longlines, which make up 86% of the total Cypriot fishing fleet. Additionally, SSF operate near the coastline and across the continental shelf, and throughout the year with higher fishing effort during spring and summer months [40].

Pilot interviews were conducted with ten fishermen in order to finalise the questionnaire, and the necessary changes/modifications were made before the final interview questions were compiled. Prior to the interviews, participants were introduced to the study and were ensured of the anonymity of the interviewee. It was clarified from the beginning that the study had no association with regulatory and fishing authorities. These were important steps to gain the trust of the interviewees as there were sensitive questions such as reporting monk seal bycatch that could have affected their responses. Furthermore, the researcher/interviewer was already known and trusted among many fishermen from previous studies and projects [39,41] which supported the process of recommending the researcher to other fellow fishermen. These steps were followed in order to minimise withholding of information and helped with the clarity and somehow the validity of their responses. The snowball sampling method was used to select the interviewees of the study [42]. This non-probabilistic sampling technique is used to interview people which have been nominated from the previous person being interviewed, based on their specific characteristics, which in this case was their experience with monk seal interactions.

The semi-structured questionnaire was divided into four parts with 46 questions in total, composed of closed-ended and open-ended questions: (Part I) demographics and vessel characteristics; (Part II) monk seal interactions in 2020; (Part III) general information on monk seal population trend; and (Part IV) fishermen overall experience/knowledge on monk seal interactions. The only two open-ended questions concerned the identification features of the damages caused to fishing gear and the preferred species depredated by monk seals. The answers were then coded and categorised. Printed maps with rectangle gridded cells of 1 × 1 km were used during the interviews to record encounter/bycatch locations of monk seals, as indicated by the participants. A simple identification guide showing the different morphological characteristics between adult males, adult females, juveniles and newborns was used during the interviews. Using the guide, fishermen were able to indicate the life stage of the individuals encountered in the last year (2020). Responses were then categorised into two morphological groups, juveniles and adults.

Standardised interview methodologies and best practices previously used in fisheries studies were followed during all interviews to maximise the clarity and consistency of the responses, e.g., [13,39,43,44,45]. The interviews were conducted in-person and private with the participant to avoid the influence of other fellow fishermen. In order to minimise bias and avoid influencing the participants, the interviewer always appeared neutral during the interviews, allowing the time needed to respond to the question without rushing to the next one. Strange responses and reliability of the information provided were recorded by the researcher, and at the end of each interview, the interviewer completed a questionnaire to assess the honesty, engagement and certainty of the participants’ answers.

The encounter rate (ER), the depredation rate (DR) and the incidental capture rate (ICR) were calculated based on the total annual fishing effort (days) of each participant interviewed and the total number of monk seal encounters, total number of days with depredation and total number of individuals incidentally captured during 2020. Therefore, the ER, DR and IR are expressed as:

ER = Total number of monk seal sightings/Total fishing effort

DR = Total number of days with depredation/Total fishing effort

ICR = Total number of individuals incidentally captured/Total fishing effort

Descriptive statistics were used (i.e., means, standard deviation and percentages) to quantitatively describe and summarise the data. A spider chart was used for the graphical representation of multivariate data. Where appropriate, the non-parametric chi-squared test was used for group comparisons. Statistical analyses were performed using the statistical software R [46]. Statistical significance was set at α = 0.05. Standard deviation is referred to as SD.

A map of monk seal encounters in 2020 was created based on participants’ indications. The participants also provided information on depth and distance from the shore at the point of the encounter, the type of interaction they had with the monk seal as well as the life stage groups of the individuals. With this information, the encounter points were then plotted as accurately as possible on Google Earth and then exported into QGIS. In order to draw conclusions and find hotspot areas of monk seal encounters, the Kernel Density Heatmap tool was used and each point was assigned a value from 1 to 3. The values were assigned by calculating the density of point features around each output raster cell, with all points being assigned a weight of one and thus counted only once. The units of the above-mentioned scale can more easily be defined as the densities of the output values which represent the predicted density value and depend on the proximity of the observed points. Lastly, using the Buffer tool, a buffer of one km perimeter was drawn around each point to compensate for any errors which might have resulted from the manual plotting of sighting points.

## 3. Results

In total, 90 interviews were conducted with professional small-scale fishermen at nine ports: Ayia Triada (*n* = 8, Famagusta region), Paralimni (*n* = 9, Famagusta region), Ayia Napa (*n* = 21, Famagusta region), Liopetri (*n* = 11, Famagusta region), Larnaca (*n* = 8, Larnaca region), Zygi (*n* = 10, Larnaca region), Limassol (*n* = 14, Limassol region), Paphos (*n* = 4, Paphos region) and Latsi (*n* = 5, Paphos region). The interviews covered 54%, 21%, 27% and 10% of the total registered vessels of categories A and B in the region of Famagusta, Larnaca, Limassol and Paphos, respectively.

### 3.1. Demographic of Small-Scale Fishermen Interviewed

The fishermen interviewed were between 29 to 94 years of age, with a mean age of 56 (SD = 11.5) years. Fishermen’s professional fishing years in activity ranged from 3 to 63 years with a mean of 25 years (SD = 14.5). Out of the 90 interviewees, 2 received no formal education (2%), 26 went to primary school (28%), 6 to lower-secondary school (7%), 50 to upper-secondary school (50%) and 6 had a university degree (7%). The majority of the participants reported that fishing is their main occupation (76%) while the rest reported that fishing is their secondary profession. For many participants (63%), fishing was their only source of income, whereas others reported having additional sources of income. The average annual fishing effort of all fishermen interviewed was 201 days (SD = 57). Lastly, only a small proportion (30%) of the interviewees reported that their family had not been related to professional fishing activities prior to them.

### 3.2. Small-Scale Fisheries-Monk Seal Interactions

#### 3.2.1. General Information on Interactions

Most fishermen (77%) reported that depredation by monk seals is affecting both trammel nets and gillnets, whereas 17% reported only gillnets are affected and 6% only trammel nets. No fisherman reported depredation on bottom set longlines. Depredation occurred more often in shallow waters (0–50 m) (82%) followed by intermediate waters (50–100 m) (3%) and deep waters (>100 m) (1%). When fishermen were asked to report which habitats are preferred by monk seals, 45% stated rocky hard bottoms, 29% that they prefer any type of habitat, 14% reported Posidonia meadows and 12% sandy-muddy bottoms. Thirty-five per cent of the participants reported that depredation by monk seals is a rare event, 25% that it is a frequent event and 24% that it sometimes happens. Lastly, a few participants reported that depredation always happens (16%), meaning that they experience monk seal depredation on every fishing trip.

Interactions between SSF and the Mediterranean monk seals could have varying consequences to fishermen, with the most common reported to be monk seals taking fish from fishing gear (depredation) (97%), causing damage to fishing gear (91%) and catch (69%). Some fishermen (37%) also reported that when monk seals are present, they scare and drive the fish away (‘catch scattering’) leading to reduced catch. A total of 60% of the damages caused to fishing gear or catch reported by fishermen were described as chewed nets with holes and strips. Some fishermen (26%) claimed that they were able to distinguish the damages caused by the monk seals from other predators like dolphins, turtles and the invasive silver-cheeked toadfish. However, others (14.1%) stated that damages caused by monk seals can only be confirmed by the presence of monk seals.

Answers from the open-ended question were coded into eight categories and the frequency of each answer is shown in Table 1. To mitigate the problem, the fishermen interviewed stated they have neither taken any measures nor do they know of any possible solutions. They expressed the belief that currently there is no effective solution to this problem.

Fishermen were then asked to report species that the monk seals prefer to depredate on. Based on fishermen’s responses, 22 species and three generic fish categories were identified from this open-ended question. The eight most common answers were Shiny fish (18%), followed by Don’t know (16%), *Boops boops* (13%), Various fish (10%), *Spicara maena* (8%), *Mullus* spp. (5%), *Diplodus sargus* (3%) and *Sparisoma cretense* (3%) (Table 2). As explained by the fishermen, shiny fish refers to fish that are silverish and shiny in colour like for instance *Boops boops*, *Diplodus sargus*, *Diplodus vulgaris*, *Spicara maena*, *Spicara smaris*, *Sphyraena* sp., *Sardina pilchardus*, *Pagellus erythrinus* and *Pagrus pagrus*.

#### 3.2.2. Monk Seals Interactions in 2020

**Figure 1:**
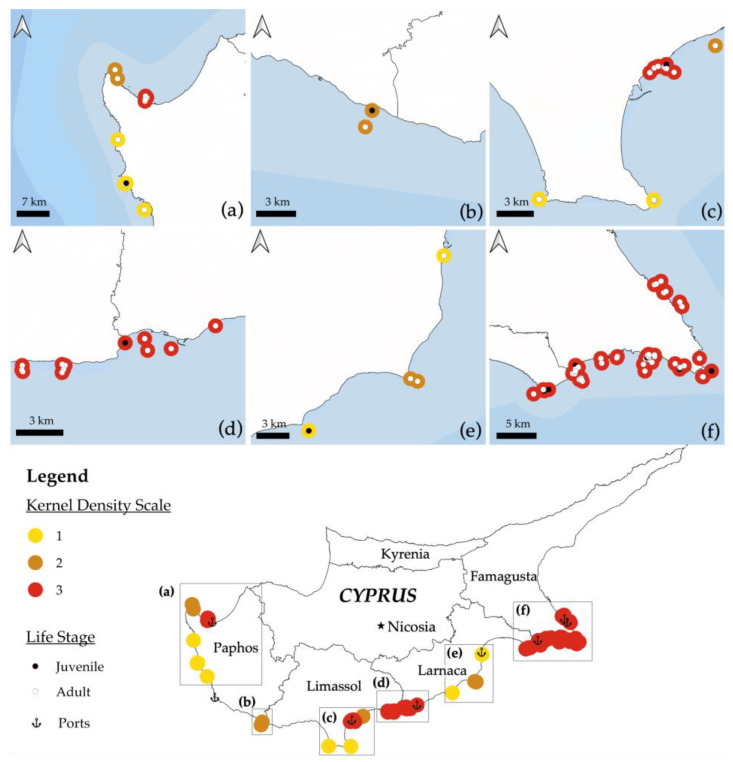
Heat map of Mediterranean monk seal encounters in 2020 based on fishermen’s knowledge with information on life stage at the time of observation. KERNEL density algorithm was used as a non-parametric spatial analysis method to present approximate distribution of the observed species in Cyprus and the probability density per 1 km radius. Panels (**a**–**f**) are a magnification of the rectangles (**a**–**f**) shown on the map of Cyprus. Interviews were conducted at the ports indicated on the map of which two overlap in the Famagusta region. The analysis was performed using QGIS.

Mediterranean monk seal encounters ranged from 0 to 15 with a mean of 2 (SD = 2.6) per fisherman and with a total of 170 encounters in 2020. According to fishermen’s observations, the monk seal encounter rate was calculated at 0.01 (0.95% of fishing trips). In total, 43 locations have been identified for monk seal encounters, in the districts of Famagusta (*n* = 18), Larnaca (*n* = 6), Limassol (*n* = 11) and Paphos (*n* = 8) (Supplementary Table S1). In descending order, the most monk seal encounters were recorded in the district of Famagusta followed by Limassol, Paphos and Larnaca. The spatial distribution of monk seal encounters is shown in Figure 1.

**Figure 2:**
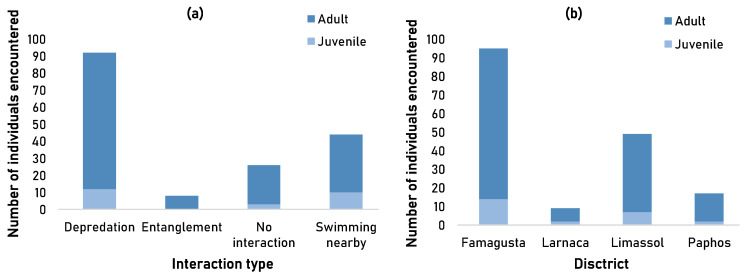
Number of Mediterranean monk seals encountered in 2020 based on fishermen’s observations in relation to (**a**) the interaction typology and (**b**) location.

Monk seal encounters with SSF were tested for correlation to life stage groups and typology of interaction during an encounter. The majority (85%, *n* = 145) of the monk seals encountered in 2020 were adult individuals. Overall, fishermen reported depredation by the monk seal as the primary outcome when encountered (54%, *n* = 92), followed by monk seal swimming nearby the boat or fishing gear (26%, *n* = 44), no interaction (15%, *n* = 26) and entanglement in fishing gear (5%, *n* = 8) (Figure 2a). No significant correlation was detected between the two variables, indicating that interaction type and life stage groups (adult, juvenile) of monk seals are in fact independent from each other (χ^2^ = 5.87, df = 6, *p*-value = 0.44). Depredation events were higher in Famagusta (*n* = 49), followed by Limassol (*n* = 27), Paphos (*n* = 9) and Larnaca (*n* = 7). Incidental entanglement was only recorded in Famagusta (*n* = 8).

Fishermen who reported monk seal entanglement/incidental capture on fishing gear clarified that the animal escaped while the nets were being towed. In all cases of incidental capture, the animals were entangled only on trammel nets (100%) and by their hind flippers. The majority of fishermen (75%) reported that monk seals cause damage to fishing gear and catch whilst the rest reported that they do not cause any damages and if they do cause damage, it is insignificant compared to other taxa. The incidental capture rate and depredation rate of monk seals were calculated at 0.0004 (0.04% of fishing trips) and 0.005 (0.51% of fishing trips), respectively.

More than half of monk seal encounters were recorded in Famagusta (56%, adult = 81, juvenile = 14) and about a third of encounters were recorded in Limassol (29%, adult = 42, juvenile = 7). Larnaca had the lowest monk seal encounter records (5%, adult = 7, juvenile = 2). Similar to the interaction type, no significant correlation was detected between the two variables, indicating that location and life stage groups of monk seals are in fact independent from each other (χ^2^ = 4.62, df = 6, *p*-value = 0.59) (Figure 2b). 

**Figure 3:**
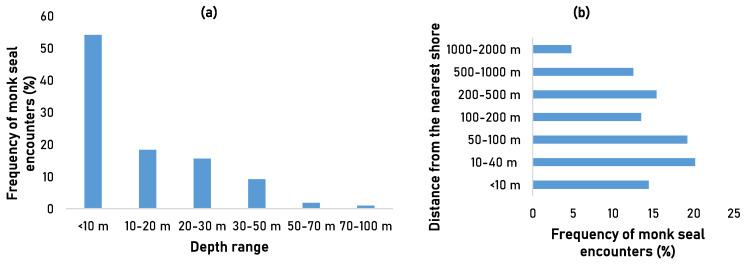
Frequency of monk seal encounters per (**a**) depth (m) and (**b**) distance from the nearest shore (m) in 2020 based on fishermen’s observations.

Most monk seal encounters, including depredation events, were reported during spring and summer (28% and 26%, respectively) and less during winter and autumn (18% and 9%, respectively). Some fishermen reported encounters with monk seals throughout the year (19%). In terms of depth, most monk seal encounters were recorded at less than 10 m depth (54%). More than half (54%) of monk seal encounters were recorded at less than 100 m distance from the nearest shore and about 95% of the encounters were recorded in less than a km (Figure 3).

**Figure 4:**
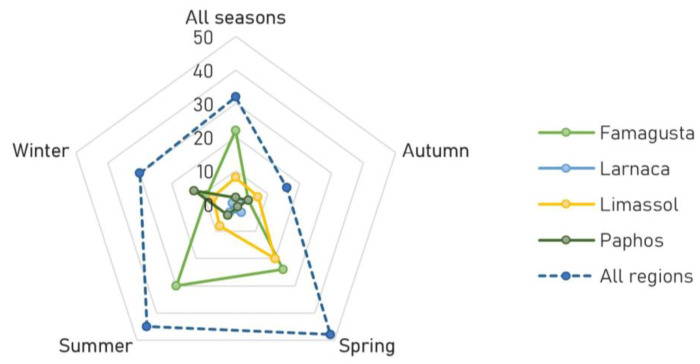
Graphical representation of the number (chart scale) of Mediterranean monk seals encountered in 2020 based on fishermen’s observations in relation to the region and season observed.

The findings also indicated that, in terms of seasonal and spatial distribution, the Famagusta region had the highest number of monk seal encounters during spring and summer (50% and 67%, respectively). The Limassol region accounted for the majority of encounters during autumn (44%), while the Paphos region had the highest number of encounters during winter (43%) (Figure 4).

### 3.3. Trends of the Mediterranean Monk Seal Population

**Figure 5:**
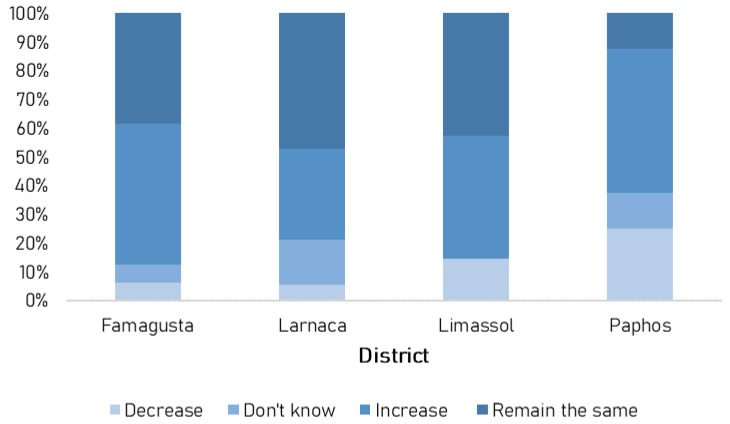
Frequency of responses per district with regards to Mediterranean monk seal population changes in the last 10 years.

Finally, fishermen were also asked to report if the population of monk seals in their area has increased, decreased or remained the same in the last ten years. More than a third (44%) replied that the population has increased, 39% that the population has remained the same, 9% that it has decreased and 8% did not know (Figure 5).

## 4. Discussion

The current study aims to provide information and improve our understanding of the interactions between the Mediterranean monk seal and SSF in the Republic of Cyprus through the fishermen’s ecological knowledge (FEK). Altogether, our findings show that fisheries’ interactions with monk seals and their following consequences are considered minimal compared to other taxa [26,38,39], which are consistent with what was previously reported in SSF in the Eastern Mediterranean [33]. The findings also revealed that fishing gear, depth and seasonality are the main drivers leading to interactions with monk seals. This is the first study to assess the interactions between monk seals and SSF in Cyprus.

Although small-scale fisheries by definition could be considered minor to an extent, on the contrary, they are of a large-scale economic and socio-cultural importance for coastal communities. The Mediterranean SSF employs 59% (~115,000 people) of the total employment in Mediterranean fisheries, comprises 82% of the total Mediterranean fishing fleet and generates a revenue of USD 0.78 billion (27% of the total revenue generated in Mediterranean fisheries) [51]. Despite their importance on regional and national levels, small-scale fishermen generate a very low profit margin, making them extremely vulnerable to challenges such as boat repairing, injuries, pandemics, economic crises and politics, reduced fish stocks and fishing grounds, climate change, non-native species and interactions with marine megafauna [51]. Therefore, even a small reduction in their profits, caused for instance by marine megafauna depredation, could have a significant negative economic impact on them [52,53,54].

Similar to our findings, studies showed that the sector is aging with the majority of small-scale fishermen being above the age of 40 while younger generations avoid entering the profession [33,51,52]. The majority of fishermen in our study reported that fishing is their only occupation and sole source of income, and more than half of them finished high school, which is in accordance with SSF demographic characteristics reported in Greece [33,52]. Taking into account the very limited operational and financial capacity of the sector, it is important that management actions to mitigate the interactions with the Mediterranean monk seal are supported by financial incentives and subsidies. Such management actions may include investments in technological equipment and gear modifications, as well as restrictions on temporal and spatial closed areas [31].

Overall, the highest monk seals encounters and depredation events were recorded in spring and summer, in shallow waters (0–50 m depth) and close to the coastline, similar to the findings previously reported in Greek SSF [33,50]. Previous findings from experimental surveys showed that the deeper the fishing gear was deployed, the lower the probability of damages and depredation by monk seals [50].

Dietary analysis confirms that the diet of the Mediterranean monk seal is comprised primarily from coastal species [47,48,49], as shallow waters and their associated habitats are important foraging, breeding and nursery areas for many fish species [55,56]. Spring is an important reproductive period for a variety of fish, both demersal and pelagic species, which could migrate to shallower areas to reproduce. For this reason, these species are more accessible to SSF [57] and to monk seals, potentially explaining the seasonal and bathymetric trend observed in the fishermen’s responses of the present study. Therefore, it is likely that the SSF and the monk seals are exploiting the same biological resource which may create an antagonistic environment between them, also known as biological interaction [58]. Biological interactions may lead to operational interactions (physical interaction with fishing gear and catch) such as depredation [59]. However, it is important to mention also that spring and summer are the seasons with the highest fishing effort [40], indicating that the likelihood of interaction with monk seals might be higher.

Even though depredation has been reported as an occasional event, it was however the most common interaction type with monk seals, being responsible for causing damage to fishing gear and catch [33,50]. In this study, some fishermen have reported monk seal depredation as a very frequent event (41%); however, considering the very low values of ER, DR and ICR as well as our prior experience with the fishing sector in Cyprus, we consider this as an unlikely event. Such intense responses may be the result of frustrated fishermen, stemming from the various challenges they encounter in their profession. Another biological interaction that has been reported by the fishermen of this study is catch scattering, which means the act where predators reduce catch efficiency by driving fish away from the fishing ground. Such a form of biological interaction has been previously reported in marine mammals-fisheries interaction studies [33,54,60]. This type of interaction might be often misidentified as depredation and confused with the animal’s direct predation on wild fish [61]. Fishermen reported being able to distinguish the damages caused to fishing gear due to depredation by the monk seal. This is extremely difficult to verify and/or quantify, often requiring a large number of onboard observations and hence, financial resources. Surprisingly, though, identical damage patterns on nets have been previously described by fishermen from Greece [33]. Such information may support future efforts in identifying and quantifying the damages caused to fishing gear by monk seals.

The findings of this study also provide insights into the Mediterranean monk seal preference on species prey predation. The monk seal feeds on different coastal and pelagic fish species, preferably from the Sparidae family and on different cephalopods and crustacean species [47,48,49,62], some of which have been reported from the fishermen in the current study. To the best of our knowledge, the fish species *Sphyraena* spp., *Siganus* spp., *F. commersonii*, *S. dumerili*, *S. pilchardus*, *S. aurata* and the crustacean *S. latus* have never been reported before in the diet of the Mediterranean monk seal. More interesting, though, are the records of the two non-indigenous species, *Siganus* spp. and *F. commersonii.* However, this does not necessarily mean that these species are part of the natural feeding habits of the animal. Catch removal from fishing gear is an opportunistic feeding behaviour through which, according to the Optimal Foraging Theory, an animal makes foraging decisions according to prey type and availability with the aim to gain more energy from feeding versus the energy expended in capturing the prey item [63]. Hence, prey selection and foraging mode of animals can change in relation to food availability [64]. Opportunistic feeding behaviour has been previously reported in numerous studies for marine mammals and sharks, e.g., [65,66]. In an ultra-oligotrophic environment like Cyprus where biological productivity is very low compared to the central and western Mediterranean ([67], and references therein), such foraging decisions are likely to happen especially for generalist predators like the Mediterranean monk seal [62].

Trammel nets and gillnets are the primary gears used in Cypriot SSF which are also the most widespread fishing gears used (80%) among SSF in the Mediterranean [68]. According to official government data, trammel nets are slightly more often used (57%) compared to gillnets in Cyprus. Trammel nets are designed to catch a high variety of species and are commonly used when there is no specific target catch, which makes them less selective compared to gillnets. In line with the results observed in Greece, interactions with monk seals affected both trammel nets and gillnets (77%), but compared between the two fishing gears, a greater number of fishermen reported incidents involving only gillnets [33,50]. This may be due to the fact that gillnets are often used to specifically target certain species such as *B. boops*, *Spicara* spp., *Diplodus* spp., *Mullus* spp. and *Pagellus* spp., which are among the species previously reported in the diet of monk seals [47,48,49,62]. Moreover, all cases of accidental entanglement documented in the present study involved trammel nets. This is likely because trammel nets are more intricate fishing gear compared to gillnets, consisting of three layers of netting as opposed to a single layer in gillnets.

Compared to the results from Greece [33,50], the encounter, depredation and incidental capture rates have been found to be quite low. This is most likely due to the relatively small monk seal population size of about 20 individuals in Cyprus, which indicates that the interactions with fisheries are expected to be considerably less than those in Greece. However, fishing efforts, rate of encounters and seals’ population should be weighted with other areas in order to have comparable values. Nevertheless, the incidental capture of eight individuals in 2020 recorded in the current study is still alarming. Although there have been reports of intentional killing of monk seals by fishermen in Greece [62], the encouraging finding of this study is that none of the fishermen exhibited any hostility or negative attitude towards the monk seals. During the interviews, Cypriot fishermen were repeatedly referring to the monk seals as ‘our friend at sea’. However, this might not always be the case.

The higher numbers of monk seal encounters of both adult and juvenile individuals were recorded in Famagusta and Limassol regions, very likely related to the fact that these are the only regions with suitable pupping caves, including Akamas Peninsula (Paphos) [69,70]. During the breeding period between September–December of 2019, three pups were born in the regions of Limassol and Akamas [71] and in November–December 2021 two pups were born in Ayia Napa (Famagusta) [72]. As expected, Larnaca was the region with the lowest SSF-monk seal interactions, probably related to the fact that no resting or pupping caves are available in the area and hence, the coastal use of this specific area by monk seals may be more limited [7]. This study also revealed a longitudinal movement of the species between the different seasons. It seems that monk seals move from the western to the eastern region of Cyprus during the summer months (Figure 1). However, for such an assumption to be verified, it has to be supported with scientific monitoring surveys. In regard to the species population trend, most fishermen stated that the monk seal population has risen over the past decade, which aligns with scientific evidence indicating that the species has been recovering in Cyprus since 2011 [7,71].

This study also highlights the value of well-designed interview-based studies and the incorporation of FEK [73] in fisheries science. Studies based on FEK are practical in obtaining useful information where resources and data on a particular topic are lacking [74,75,76,77]. Until very recently, fishermen’s knowledge has been ignored by scientists and policy- and decision-makers [78]. Studies have shown that fishermen FEK contributed to filling knowledge gaps in species’ diets, ecology and habitats [76,77,79,80]. Similarly, the fishermen in the present study provided relevant ecological knowledge on the monk seal diet and habitats as well as the species’ behaviour in relation to fisheries. Finally, FEK is also important in supporting and improving the political position and collaboration of fishermen and hence, contribute to the long-term successful implementation of management measures [81,82]. However, despite the many merits of this approach, such information should be always carefully treated and interpreted and when possible, supported by conventional scientific studies [77].

This study highlights the minimal interactions between the Mediterranean monk seals and SSF in the Republic of Cyprus. Accidental entanglement of monk seals on set nets has been shown to be a possibility but with low probability. Based on the results from the current study, specific management actions are proposed. Management recommendations are as follows: (1) spatiotemporal closed areas—static set nets to be set at greater depths (>20 m depth) during spring and summer, specifically at areas with monk seal breeding caves, for example in Ayia Napa, Limassol and Akamas, and (2) run awareness campaigns for the public, stakeholders and sea users regarding the Mediterranean monk seal, and educational workshops specifically designed for fishermen on how to safely handle and release a monk seal in the case of accidental entanglement.

## 5. Conclusions

In data-scarce topics, FEK has been shown to be an important and valuable as well as complementary tool in gaining basic and advanced information on different levels of species and community ecology. Understanding the vast amount of knowledge that fishermen have and the vulnerability and social and economic importance of the sector, we suggest that fishermen should actively participate in fisheries science. Following the principles of the bottom-up approach to fisheries management, fishermen should be involved in the decision-making process and contribute to the development of fisheries management measures. The current work contributes also to the wider context of SSF research and to the conservation of the monk seal in the Mediterranean Sea. Currently, many different actions have been led and successfully implemented by local authorities to support the conservation of the species in Cyprus including population monitoring, the clean-up and restoration of caves and the establishment of dedicated marine protected areas. Finally, we suggest that future research efforts should focus on understanding and evaluating the impacts of the physical disturbance and noise pollution caused by other anthropogenic stressors in this highly touristic area.

## Figures and Tables

**Table 1:** Coded responses of the open-ended question regarding the identification features of the damages caused to fishing gear and catch by the Mediterranean monk seal. The number of times and frequency of each coded response are indicated in the table.

Identification Feature	*N*	%
Type of damage on nets and holes different from other predators	1	1.2
Fish head is missing	2	2.4
It cannot be determined	2	2.4
Depredated fish and damaged nets with rounded holes	5	5.9
Presence of a monk seal	12	14.1
Holes on nets smaller and more rounded than from dolphins	17	18.9
Chewed nets with large holes and strips	51	60

**Table 2:** List of species and categories of fish that monk seals prefer to feed/depredate on based on fishermen’s responses. The number of times and frequency of each response are indicated in the table together with information on species origin and if reported in previous studies. Cross (+) indicates yes.

Species and Categories	*N*	%	Indigenous Species	Previously Reported [47,48,49,50]
**Osteichthyes**				
*Boops boops*	18	13.3	+	+
*Diplodus sargus*	4	3.0	+	+
*Diplodus vulgaris*	2	1.5	+	+
*Mullus* spp.	7	5.2	+	+
*Pagellus erythrinus*	1	0.7	+	+
*Spicara maena*	11	8.1	+	+
*Spicara smaris*	1	0.7	+	+
*Sphyraena* sp.	2	1.5	+	
*Siganus* spp.	1	0.7		
*Oblada melanoura*	2	1.5	+	+
*Fistularia commersonii*	1	0.7		
*Seriola dumerili*	2	1.5	+	
*Sardina pilchardus*	1	0.7	+	
*Sparisoma cretense*	4	3.0	+	+
*Dicentrarchus labrax*	2	1.5	+	+
*Dentex dentex*	1	0.7	+	+
*Pagrus pagrus*	1	0.7	+	+
*Sparus aurata*	3	2.2	+	
**Cephalopoda**				
*Sepia officinalis*	3	2.2	+	+
*Loligo* sp.	2	1.5	+	+
*Octapus vulgaris*	3	2.2	+	+
**Crustaceans**				
*Scyllarides latus*	1	0.7	+	
**Species categories**				
*Various fish*	14	10.4		
*Shiny fish*	24	17.8		
*Non-venomous fish*	2	1.5		
**Don’t know**	22	16.3		

## Data Availability

Data are available upon reasonable request to the authors.

## Supplementary Materials

The following supporting information can be downloaded at: https://www.mdpi.com/article/10.3390/ani13132164/s1, Table S1: Locations reported with encounters of the Mediterranean monk seal in 2020.
