# Supplementary material for: Understanding the Interactions between Small-Scale Fisheries and the Mediterranean Monk Seal Using Fishermen’s Ecological Knowledge
Source: Animals (Basel). 2023 Jun 30;13(13):2164. doi: 10.3390/ani13132164 (PMC10339921; doi:10.3390/ani13132164)
Supplement: Supplementary file 1 [file animals-13-02164-s001.zip › animals-2405650-supplementary.pdf]

Table S1: Locations reported with encounters of the Mediterranean monk seal in 2020.

Location	Times Reported	%	District
Aquaculture units, Potamos, Liopetri	11	9.0	Famagusta
Ayia Napa port	23	18.9	Famagusta
Ayia Thekli, Sotira	3	2.5	Famagusta
Ayia Triada port, Paralimni	3	2.5	Famagusta
Cape Greco, Ayia Napa	2	1.6	Famagusta
Cape Pyla, Xylophagou	3	2.5	Famagusta
Kaminoudia, Paralimni	1	0.8	Famagusta
Kapparis, Paralimni	3	2.5	Famagusta
Kermia, Ayia Napa	3	2.5	Famagusta
Konnos, Protaras	1	0.8	Famagusta
Kryo Nero, Ayia Napa	3	2.5	Famagusta
Makronissos beach, Ayia Napa	3	2.5	Famagusta
Liani, Xylophagou	2	1.6	Famagusta
Paralimni	1	0.8	Famagusta
Pernera, Paralimni	2	1.6	Famagusta
Potamos port, Liopetri	2	1.6	Famagusta
Sea caves, Ayia Napa	2	1.6	Famagusta
Teloni, Liopetri	1	0.8	Famagusta
Larnaca port	1	0.8	Larnaca
Petounda, Mazotos	1	0.8	Larnaca
Pharos, Pervolia Larnaca	2	1.6	Larnaca
Vasilikos port	1	0.8	Larnaca
Vasilikos Power Station, Zygi	2	1.6	Larnaca
Zygi port	1	0.8	Larnaca
Ayios Georgios Alamanou	4	3.3	Limassol
Dasoudi beach, Limassol	2	1.6	Limassol
Governor's beach, Limassol	4	3.3	Limassol
Karnayio, Limassol	2	1.6	Limassol
Limassol Marina	5	4.1	Limassol
Lianokaos, Limassol	1	0.8	Limassol
Moni, Limassol	3	2.5	Limassol
Miramali, Limassol	1	0.8	Limassol
Old port Limassol	3	2.5	Limassol
Pharos, Akrotiri Limassol	5	4.1	Limassol
Radar, Akrotiri Limassol	2	1.6	Limassol
Anassa, Latsi	4	3.3	Paphos
Cape Arnaoutis, Akamas	1	0.8	Paphos
Fontana, Latsi	1	0.8	Paphos
Karavopetres, Paphos	1	0.8	Paphos
Lahania, Petra Romiou, Paphos	1	0.8	Paphos
Petra tou Romiou, Paphos	1	0.8	Paphos
Potima, Paphos	1	0.8	Paphos
Thalassines spilies, Pegeia	3	2.5	Paphos
